# Ectopic epipericardial fat necrosis: a case report

**DOI:** 10.1186/s40792-024-01859-0

**Published:** 2024-03-08

**Authors:** Ryusei Yoshino, Masaki Nakatsubo, Nanami Ujiie, Akane Ito, Nana Yoshida, Naoko Aoki, Masahiro Kitada

**Affiliations:** 1 Department of Thoracic Surgery and Breast Surgery, Respiratory Center, Asahikawa Medical University Hospital, 2-1-1-1 Midorigaoka Higashi, Asahikawa-Shi, Hokkaido, 078-8510 Japan; 2 Department of Diagnostic Pathology, Asahikawa Medical University Hospital, 2-1-1-1 Midorigaoka Higashi, Asahikawa-Shi, Hokkaido, 078-8510 Japan

**Keywords:** Computed tomography, Epipericardial fat necrosis, Fever, Magnetic resonance imaging, Pain

## Abstract

**Background:**

Epipericardial fat necrosis (EFN) is a rare disease in which local inflammation and necrosis occur in the adipose tissue surrounding the heart, particularly epicardial fat. Few cases of EFN in which surgical resection was performed have been reported. We report a case of EFN after surgical resection of a right extrapulmonary tumor, in which a malignant disease could not be excluded.

**Case presentation:**

A 75-year-old male patient presented with fever and chest pain. A contrast-enhanced computed tomography scan of the chest revealed a lesion, 53 × 48 mm in size, with mixed fatty density spanning the middle and lower lobes of the right lung. Thoracic magnetic resonance imaging (MRI) revealed a mass with mixed fat and soft tissue density in the same area; the lesion was contiguous with pericardial fatty tissue. The tumor was diagnosed as a liposarcoma or teratocarcinoma based on imaging results; however, the possibility of lung cancer could not be excluded. Finally, EFN was diagnosed based on the postoperative histopathological examination. The patient underwent surgical resection of the suspected right extrapulmonary tumor. The intraoperative findings revealed a mediastinal mass contiguous with pericardial fat located between the middle and lower lobes. Intraoperative pathological examination of the lesion was performed using a needle biopsy; however, no definitive diagnosis was made. The tumor may have invaded the middle lobe of the right lung, and partial resection of the right lower lobe was performed in addition to resection of the middle lobe of the right lung. The patient was followed up every 3 months without adjuvant therapy. No recurrence was reported at 1 year after surgery.

**Conclusion:**

EFN should be considered in the differential diagnosis of an extrapulmonary tumor when continuity with the pericardial space is observed on MRI or other imaging studies. Surgical resection is useful in the diagnosis and treatment of EFNs. Preoperative three-dimensional reconstructive imaging and MRI should be used to identify vascular structures and confirm the continuity of the lesion with the surrounding tissues to ensure safe and rapid tumor removal.

## Background

Epipericardial fat necrosis (EFN) is a rare disease characterized by localized inflammation and necrosis of the adipose tissue surrounding the heart, especially the epicardial fat. This unique condition was first described in 1957, and there have since been few reports [1, 2]. Few cases of EFN have been reported in which surgical resection was performed and histopathological examination led to a diagnosis.

Although the pathophysiology of EFN remains unclear, the spontaneous rupture of adipocytes within epicardial fat has been implicated [3]. This releases inflammatory substances and subsequently causes inflammation of the epicardium [4]. However, the exact cause of fat necrosis remains unclear.

Owing to its rarity and lack of specific clinical and radiological features, EFN can easily be misdiagnosed, leading to unnecessary invasive procedures and delays in appropriate treatment. Therefore, it is important to increase the awareness of this disease among clinicians and radiologists to enable its early and accurate diagnosis [2].

In this report, we describe the case of a 75-year-old male patient who underwent surgical resection of a right extrapulmonary tumor for which malignant diseases, including lung cancer, could not be ruled out preoperatively, leading to the diagnosis of EFN. The purpose of this case report was to discuss the clinical, radiological, and pathological findings, contribute to the existing literature on the relatively rare condition of EFN, and emphasize the importance of considering EFN in the differential diagnosis of extrapulmonary tumors.

## Case presentation

The patient was a 75-year-old male. The patient presented to the Department of Respiratory Medicine with fever and chest pain. Imaging examination revealed a tumor outside the right lung; because malignant disease could not be ruled out, the patient was referred to our department for surgical resection of the lesion.

No relevant family history was observed. He had smoked 20 cigarettes per day for 45 years and had a Brinkman index score of 900. The patient had comorbidities, such as hypertension, dyslipidemia, and chronic kidney disease. The patient characteristics on admission were as follows: height, 164 cm; weight, 68 kg; body mass index, 25.3 kg/m^2^. Physical examination revealed no abnormal chest respiratory or cardiac sounds. No enlarged cervical lymph nodes were observed. On admission, there were no abnormal findings in blood counts, such as white blood cell counts or hemoglobin levels. Biochemical analysis revealed mildly elevated aspartate aminotransferase (54 U/L), alanine aminotransferase (68 U/L), blood urea nitrogen (20.4 mg/dL), and creatinine levels (1.09 mg/dL). Coagulation showed a mildly elevated D-dimer level of 2.20 μg/mL, but no other abnormal findings were observed. The tumor markers carcinoembryonic antigen (2.5 mg/mL), carbohydrate antigen 19-9 (9 U/mL), cytokeratin fragment antigen (1.60 ng/mL), and squamous cell carcinoma (1.4 ng/mL) were within the reference range, while the neuron-specific enolase (14.90 ng/mL) and serum soluble interleukin 2 receptor (580 U/mL) levels were slightly elevated. Respiratory function tests and electrocardiography revealed no abnormalities.

**Fig. 1:**
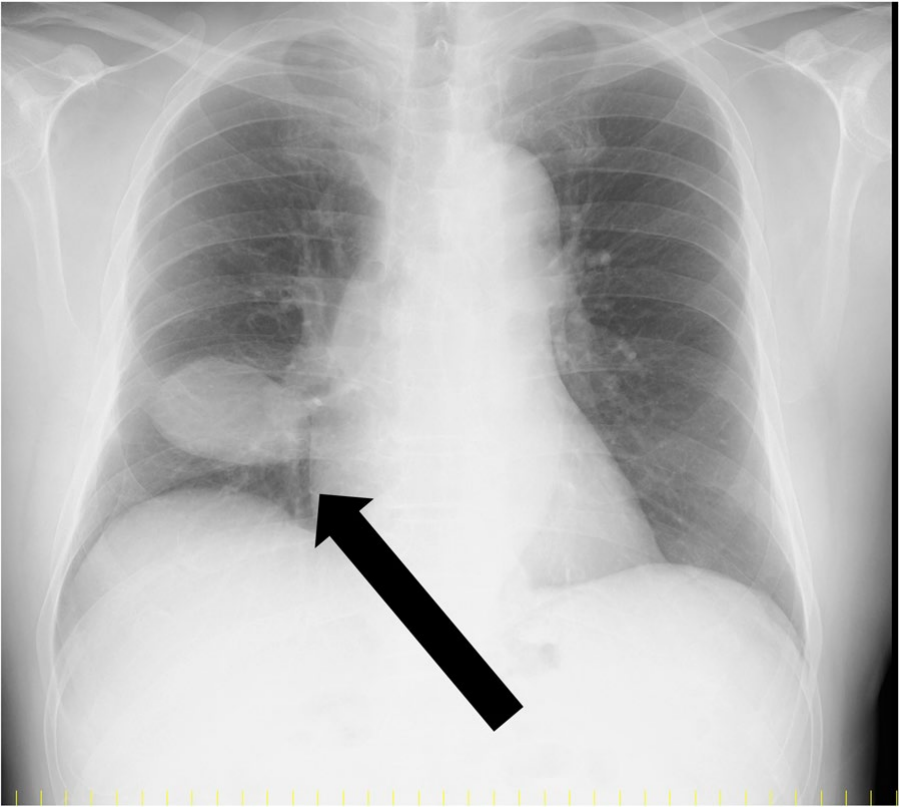
Chest radiograph (frontal view). Mass shadow in the middle and lower lobes of the right lung

**Fig. 2:**
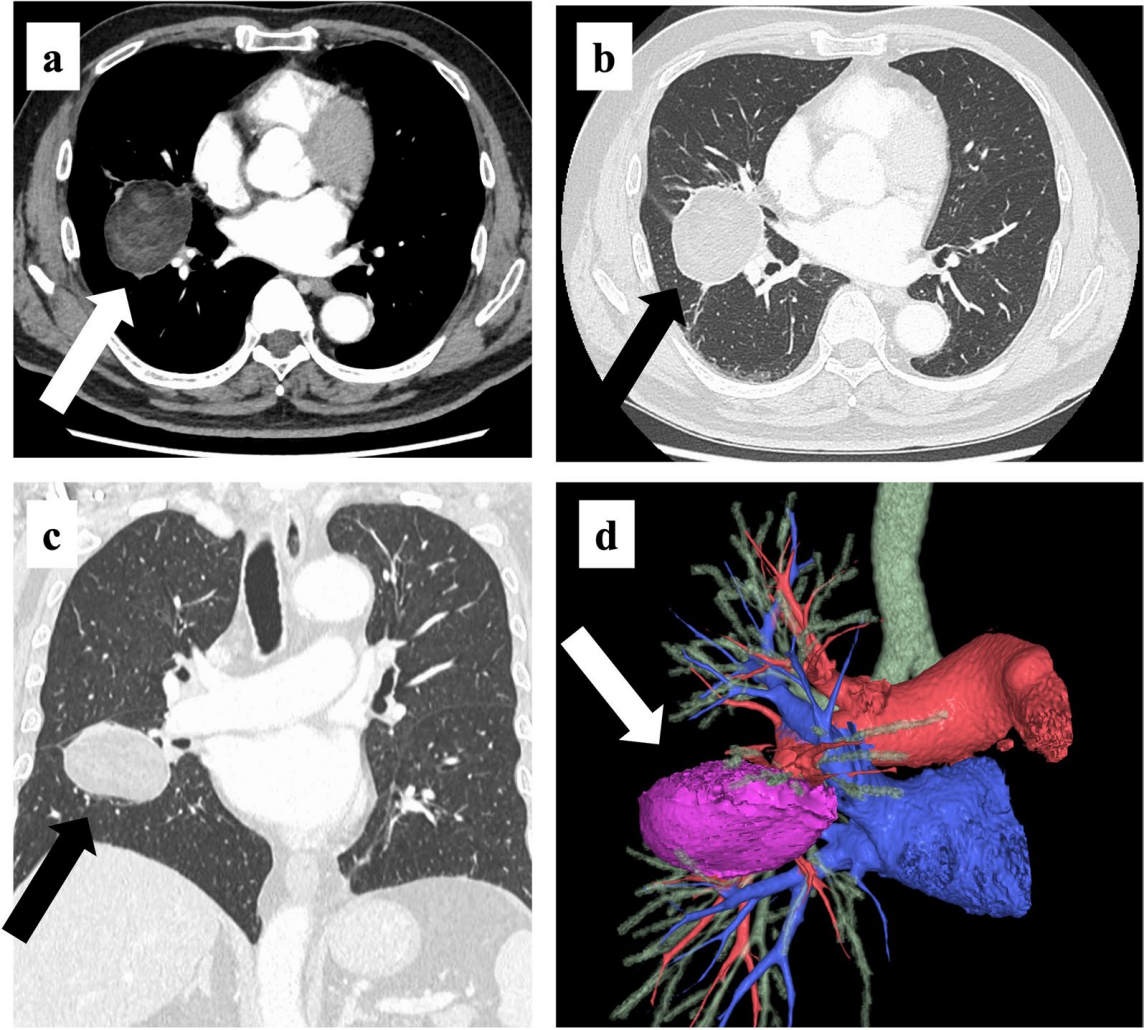
Chest computed tomography (CT) findings. **a**–**c** A 53 × 48-mm mass lesion with mixed fatty density can be seen spanning the middle and lower lobes of the right lung. **d** Three-dimensional (3D) reconstruction showing a mass lesion spanning the middle and lower lobes of the right lung with a normal bronchial branch

**Fig. 3:**
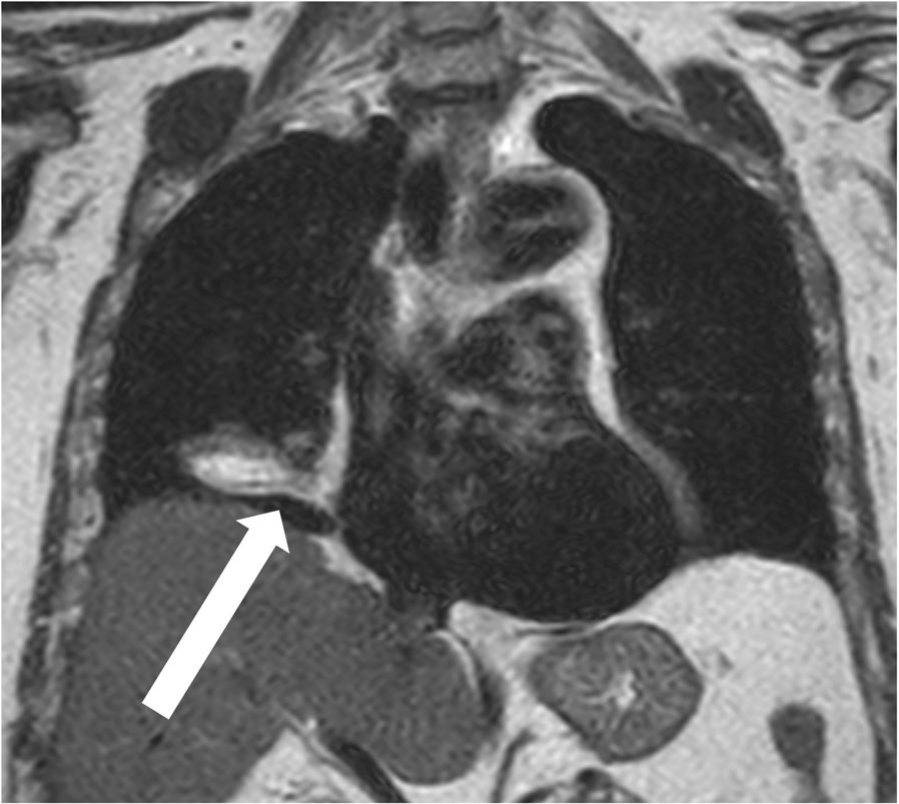
Chest magnetic resonance imaging (MRI) findings. A mass with mixed fat and soft tissue density can be observed in the major fissure area between the middle and lower lobes of the right lung. The lesion appears to be contiguous with pericardial fatty tissue

**Fig. 4.**
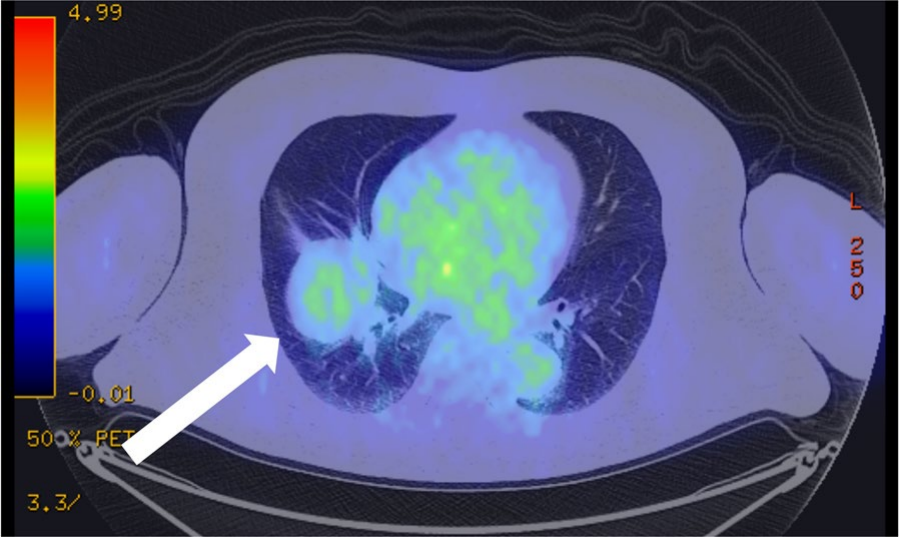
^18^F-fluorodeoxyglucose positron emission tomography findings. A 53 × 47-mm-large mass near the right pulmonary hilum, with mild accumulation of maximum standardized uptake value of 2.4

Chest radiography revealed mass shadows in the middle and lower lobes of the right lung (Fig. 1). Contrast-enhanced computed tomography (CT) of the thorax and abdomen showed a mass lesion of 53 × 48 mm in size with mixed fat density spanning the middle and lower lobes of the right lung (Fig. 2a–c). Radiologic reading revealed a mass with mixed fat and soft density in the major fissure area between the middle and lower lobes of the right lung, and the possibility of mediastinal origin was considered. No inflammation or necrosis was noted. The mass appeared to be mediastinal in origin, and liposarcoma or malignant tumors were suggested as differential diagnoses. Three-dimensional (3D) reconstruction showed a normal bronchial branch and a mass spanning the middle and lower lobes of the right lung (Fig. 2d). No significant enlargement of mediastinal lymph nodes was observed. No obvious neoplastic lesions were observed in the other organs. Thoracic magnetic resonance imaging (MRI) scan showed a mixed fatty and soft mass in the major fissure area between the middle and lower lobes of the right lung (Fig. 3). Radiologic reading showed a mass with mixed fat and soft tissue density in the major fissure area between the middle and lower lobes of the right lung, and the lesion appeared to be continuous with the pericardial fatty tissue. However, the internal characteristics were accompanied by diffusion restriction, and the possibility of a malignant lesion could not be ruled out. Contrast-enhanced MRI was not performed. ^18^F-fluorodeoxyglucose positron emission tomography showed a 53 × 47-mm mass near the right pulmonary hilum with mild accumulation of maximum standardized uptake value of 2.4 (Fig. 4). Although malignant lesions could not be ruled out, benign lesions, such as teratomas and paraneoplastic tumors, were identified as differential lesions. No bronchoscopy was performed. Based on the above findings, the possibility of malignant lesions, including lung cancer, could not be ruled out, and the subjective symptoms of fever and chest pain did not improve; therefore, surgical resection was performed. Preoperative diagnosis of this case was difficult, and the patient understood the possibility of performing a middle or lower lobectomy with invasion in mind as malignant disease if intraoperative rapid diagnosis was difficult. Since the patient was relatively old and most refused to undergo reoperation due to leftover tumor, he requested a wide resection if the possibility of malignancy could not be completely ruled out intraoperatively.

**Fig. 5:**
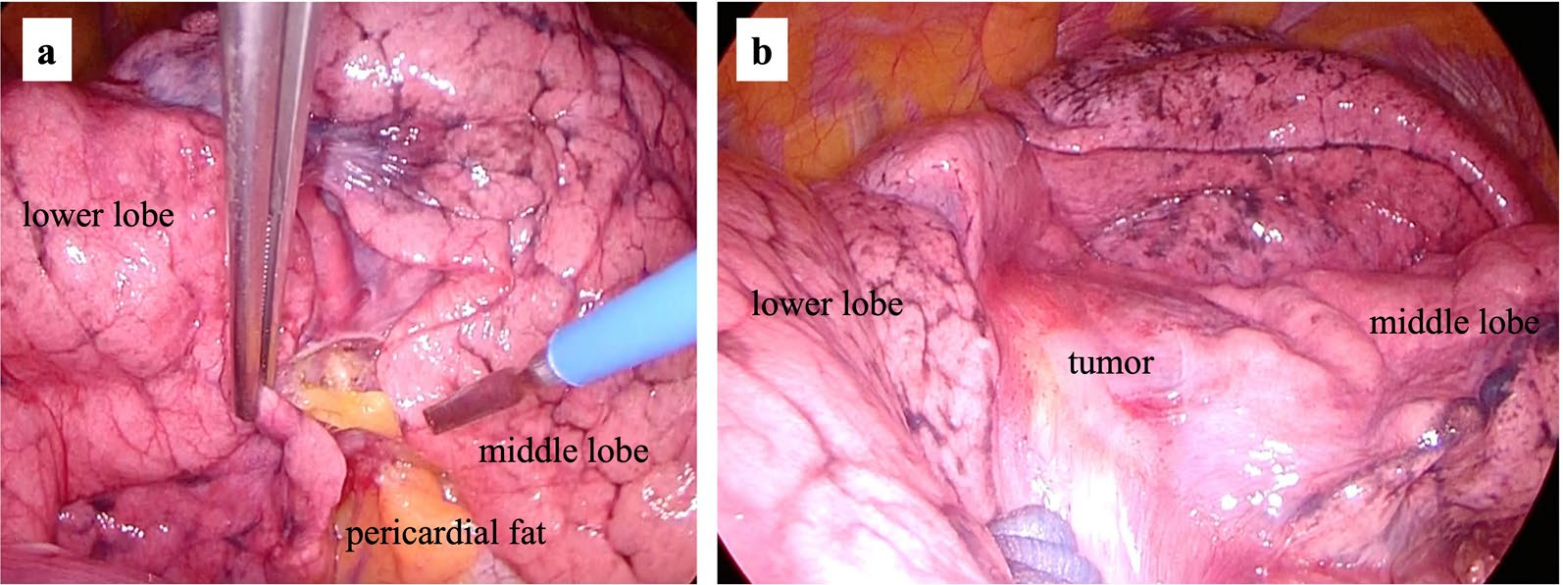
Intraoperative findings. **a** The lesion is contiguous to the pericardial fat; the contiguous pericardium was dissected for resection of the mass. **b** The lesion is located between the middle and lower lobes

At 2 months after the initial consultation, the patient underwent surgery under general anesthesia. The patient was placed in the left lateral recumbent position and a small incision was made in the right fifth intercostal space under thoracoscopic guidance. Thoracoscopy revealed no adhesions or pleural effusions. The lesion was a mediastinal pericardial fatty mass located between the middle and lower lobes (Fig. 5). There were no findings suggestive of axial torsion or ischemia of the adipose tissue. In addition, intraoperative thoracoscopic-assisted visualization did not reveal any continuity of the adipose tissue with the pericardial area or any other organ of origin that could have been inferred. Intraoperative findings did not appear that the mass was firmly adherent to the lung with inflammation. A rapid needle biopsy was performed intraoperatively for definitive diagnosis, and the result was a specimen with a high degree of histologic degenerative necrosis. Fat necrosis was also observed, as well as partial proliferation of short spindle-shaped cells. The high degree of degenerative necrosis made it difficult to estimate benign or malignant histology. The surgeon initially attempted to resect the mass alone, but because invasion into the middle lobe of the right lung and into the lower lobe of the right lung could not be ruled out, and because it was difficult to determine benign or malignant by intraoperative rapid needle biopsy, partial resection of the right lower lobe was performed in addition to tumor resection and resection of the right middle lobe. Right middle lobectomy was easily performed by preoperative 3D reconstruction to identify the vessels, the middle lobar bronchus was cut and removed after V4 + 5 was dissected, and A4 + 5 was dissected from the middle pulmonary artery trunk. Partial resection of the combined lower lobes of the right lung was performed using an automatic suturing machine. The thoracic cavity was cleaned and a leak test was performed. The operative time was 1 h 30 min, and the blood loss was 10 mL.

**Fig. 6:**
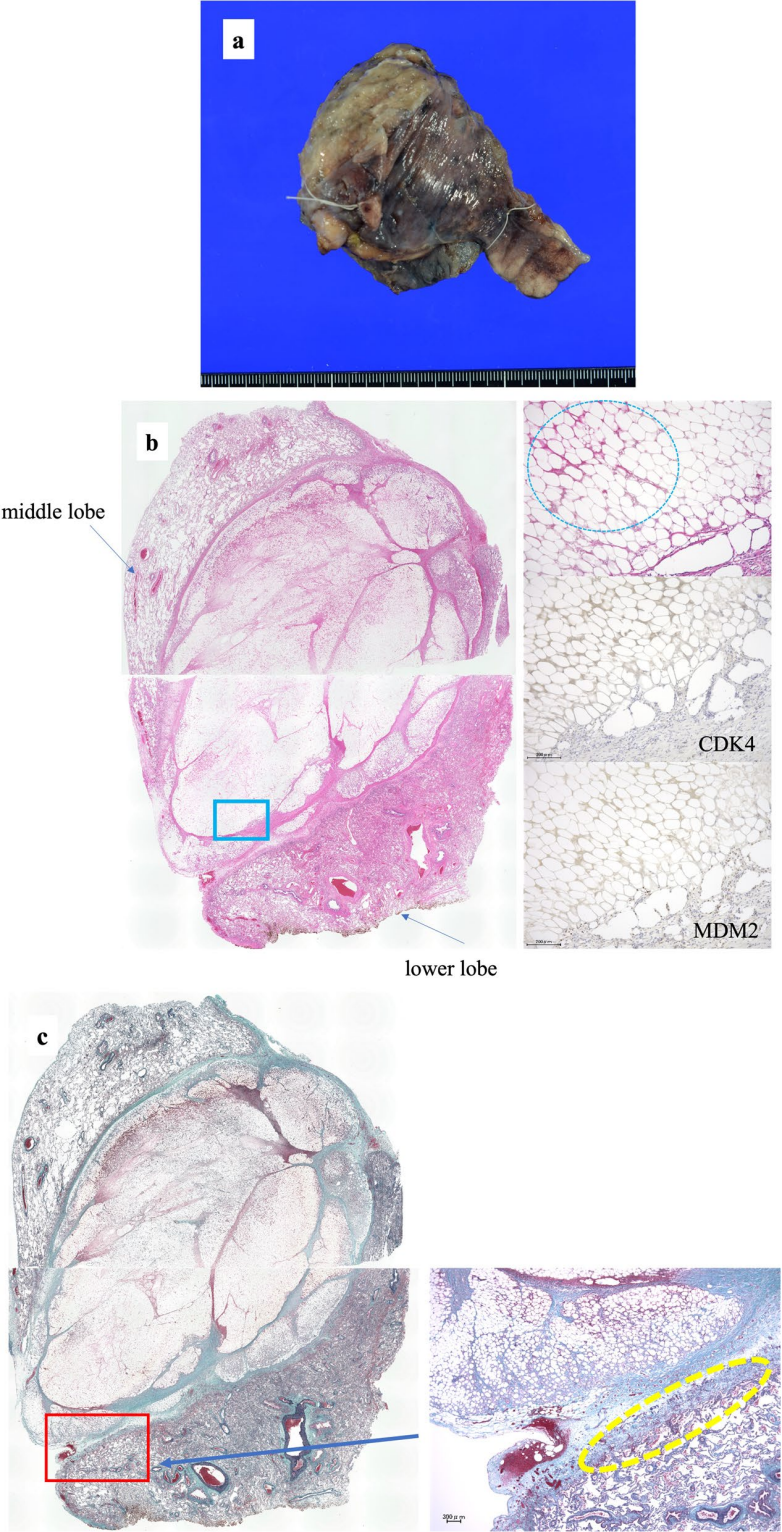
Gross and histopathological findings. **a** Gross findings of the tumor. Size, 8 × 5.5 × 3.5 cm. The split surface shows a well-defined, internally heterogeneous, full-blown mass measuring 6 × 5.5 × 2.7 cm in size. The margins are yellowish white. **b** The tumor is located between the middle and lower lobes, and the lesion is composed of adipose tissue covered by a fibrous capsule. In the blue square, the upper right adipocyte is devoid of nuclei, i.e., necrotic. The sizes of the cells are generally well aligned (hematoxylin–eosin [HE] staining, scale bar: 200 μm). There was no morphologically discernible liposarcoma or malignant findings. Immunohistochemistry shows that MDM-2 was generally negative in adipocytes and cyclin-dependent kinase 4 was negative. **c** In the red square, elastic fibers are preserved, indicating that the lesion is within the fatty tissue between the middle and lower lobes, rather than infiltrating the lung (Elastica–Masson staining; scale bar: 300 μm)

The gross appearance of the removed right lung intermediolateral lung tumor was 8 × 5.5 × 3.5 cm in size (Fig. 6a). The margins were yellowish-white. Histopathological examination revealed fatty tissue covered with a fibrous capsule. The interior of the capsule was extensively degenerated and necrotic and a small amount of viable material remained immediately below the fibrous capsule. Relatively large blood vessels were observed in the necrotic areas, whereas fat necrosis, numerous foamy histiocytes, multinucleated giant cells, lymphocytes, and neutrophils, as well as atypical mature adipose tissue and blood vessels, were observed in the viable areas. At the site of contact between the capsule and the lung tissue, Masson’s body formation and peribronchiolar metaplasia were observed in the lung tissue, which were considered to be caused by inflammation. No morphologically discernible liposarcoma or malignant findings were observed; MDM-2 were positive in macrophages but generally negative for cyclin-dependent kinase 4 (Fig. 6b, c). The most likely diagnosis was epicardial fat necrosis. Based on the symptoms of fever and chest pain, imaging findings, and histopathological examination, the diagnosis of EFN was made. The patient was followed up every 3 months without adjuvant therapy.

## Discussion

EFN causes acute chest pain and is often detected on chest CT scans [1]. The age at which EFN occurs remains unknown [4]; however, it is observed in both men and women and has been reported in children and patients with a transplantation history [5, 6]. The most common symptom of EFN is chest pain; other associated symptoms include shortness of breath, dizziness, tachycardia, and cold sweats, although fever and myalgia are not considered classic clinical symptoms [3, 7]. Barreto et al. [8] reported that EFN with cough and fever was not observed. The pathophysiology remains unclear, but hypotheses include hemorrhage owing to destruction of vulnerable vessels by Valsalva maneuvers, such as anger and breath-holding, or acute ischemia of the epicardial fat owing to tortuosity of the vascular stalk [5, 9, 10]. Another risk factor is the increase in pericardial fat due to obesity, which remains unknown because of the lack of case series [3, 5]. Although no characteristic findings have been reported on blood samples, electrocardiograms, or chest radiographs, mild increases in the C-reactive protein level, D-dimer level, and white blood cell counts were reported [11]. In recent years, treatment with nonsteroidal anti-inflammatory drugs has generally been conservative, rather than surgical resection, and improvement in symptoms is often observed within a few weeks to months [3].

CT is important for the diagnosis of EFN after acute coronary syndrome, pulmonary embolism, and other emergent diseases have been ruled out [2]. Typical CT findings include soft tissue and encapsulated fatty lesions with or without a fatty center in the epicardial fat. The periphery has a thin, dense soft tissue margin and is often round or oval [2, 4, 12]. They may also show inflammatory changes, such as thickening of the adjacent epicardium [8]. Most EFNs appear on the left side, with some reports indicating an incidence rate of 80% [3]. Several recent studies have demonstrated the usefulness of MRI. Cardiac MRI shows high signal on T1- and T2-weighted images, and fat content is confirmed by a uniform signal reduction on fat-suppressed images [3]. Fat-suppressed images are useful for evaluating signal changes in epicardial fat, with a decreased signal in the central lesion suggesting adipose tissue, and high signal lines along the adjacent pericardium and pleura indicating the margins of epicardial fat, signifying inflammatory or edematous changes [13, 14],^.^ teratomas, lipoblastomas, and liposarcomas. MRI with fat suppression may be useful for distinguishing these tumors from those that contain fat in the anterior mediastinum [3, 15]. However, it is often difficult to distinguish these tumors using imaging alone. In the present case, CT revealed a relatively large mass lesion with mixed fat concentration spanning the middle and lower lobes of the right lung. The mass appeared to be mediastinal in origin, suggesting a liposarcoma or a malignant tumor. Chest MRI revealed a mass with mixed fat and soft-tissue densities in the major fissure area between the middle and lower lobes of the right lung. However, the lesion appeared to be contiguous with the pericardial fatty tissue, and this, although atypical, was a point of differentiation for EFN.

This is a rare case in which a relatively large mass lesion was found in the right lung, and EFN was diagnosed on postoperative histopathological examination. Chest MRI showed continuity of the lesion with the pericardial fatty tissue, which led us to consider EFN. Therefore, if the EFN had been recalled, it cannot be ruled out that the mass may have shrunk in size on follow-up imaging every 4 or 8 weeks [13]. However, considering the fact that this was a large pulmonary mass found in the lung and that there was a possibility of malignant lesions, including liposarcoma and lung cancer, it was reasonable to perform early surgical resection rather than follow-up observation [8].

In this case, surgical resection was useful for the diagnosis and treatment of EFN. Generally, EFN is diagnosed and treated with antipyretic analgesics after excluding urgent diseases that cause chest pain. However, there are no reports describing the surgical treatment strategy in detail. Details on the surgical resection of a relatively large EFN, such as that in this case, are worth reporting. In this case, the possibility of tumor invasion into the middle and lower lobes was considered at the time of surgery. In some cases, tumor resection and combined resection of the middle and lower lobes were performed. Intraoperative findings did not rule out the possibility that the tumor had invaded the middle and lower lobes of the right lung, partly because it was difficult to determine benign or malignant by rapid needle biopsy. In the middle lobe, preoperative 3D reconstruction of the vessels and bronchi was sufficient to determine their locations, and the pulmonary arteries and veins were safely and routinely treated. Because the lower lobe was only partially involved, it was easy to perform partial resection using an automatic suturing machine. This procedure was performed to ensure a sufficient margin from the mass, considering the possibility of malignant disease.

The premise of this case was that preoperative diagnosis was difficult, and the patient wanted us to keep in mind that the tumor was malignant and invasive if intraoperative rapid needle biopsy was difficult. Therefore, the patient understood the possibility of performing a middle or lower lobectomy, and the patient most emphatically refused reoperation due to leftover tumor, which resulted in an extensive resection. Therefore, it is regrettable that a mesolobar resection was unnecessary as a conclusion. When we encounter similar cases in the future, we should keep in mind that preoperative diagnosis is very difficult, but careful follow-up may be possible if imaging findings show a mass lesion with continuity with the pericardial fatty tissue. It is important to avoid excessive lobectomy as in the present case. However, in relatively large tumors, as in this case, early surgical resection may be necessary if the tumor is malignant, so it is important to communicate closely with the patient to determine the best course of action.

## Conclusions

This study reports a case in which an extrapulmonary tumor was noted, liposarcoma and teratoid species were suspected preoperatively, and malignant diseases, including lung cancer, could not be ruled out; however, postoperative histopathological examination revealed a diagnosis of EFN. The presence of pericardial continuity on MRI or other imaging studies should be considered in the differential diagnosis of a large pulmonary mass. Although EFNs are generally curable with conservative treatment, surgical resection is useful for the diagnosis and treatment of relatively large lesions. When surgical resection is performed, preoperative 3D reconstructive imaging and MRI should be used to identify vascular structures and confirm the continuity of the lesion with the surrounding tissues to ensure safe and prompt tumor removal.

## Data Availability

Data sharing is not applicable to this article because no datasets were generated or analyzed in the current study.

## Abbreviations

CT: Computed tomography
EFN: Epipericardial fat necrosis
MRI: Magnetic resonance imaging
